# Understanding and optimization of hard magnetic compounds from first principles

**DOI:** 10.1080/14686996.2021.1935314

**Published:** 2021-09-15

**Authors:** Takashi Miyake, Yosuke Harashima, Taro Fukazawa, Hisazumi Akai

**Affiliations:** a Research Center for Computational Design of Advanced Functional Materials, National Institute of Advanced Industrial Science and Technology, Tsukuba, Japan; b Elements Strategy Initiative Center for Magnetic Materials, National Institute for Materials Science, Tsukuba, Japan; c Center for Computational Sciences, University of Tsukuba, Tsukuba, Japan; d Institute of Materials and Systems for Sustainability, Nagoya University, Nagoya, Japan; e The Institute for Solid State Physics, The University of Tokyo, Kashiwa, Japan

**Keywords:** Permanent magnet, rare earth, first-principles calculation, materials informatics, 40 Optical, magnetic and electronic device materials, 203 Magnetics / Spintronics / Superconductors, 401 1st principles methods, 602 Data analysis (AI, Machine learning, Data-driven analysis, Descriptor development, Structure search/identification)

## Abstract

First-principles calculation based on density functional theory is a powerful tool for understanding and designing magnetic materials. It enables us to quantitatively describe magnetic properties and structural stability, although further methodological developments for the treatment of strongly correlated 4f electrons and finite-temperature magnetism are needed. Here, we review recent developments of computational schemes for rare-earth magnet compounds, and summarize our theoretical studies on Nd_2_Fe_14_B and *R*Fe_12_-type compounds. Effects of chemical substitution and interstitial dopants are clarified. We also discuss how data-driven approaches are used for studying multinary systems. Chemical composition can be optimized with fewer trials by the Bayesian optimization. We also present a data-assimilation method for predicting finite-temperature magnetization in wide composition space by integrating computational and experimental data.

## Graphical abstract

**Figure:**
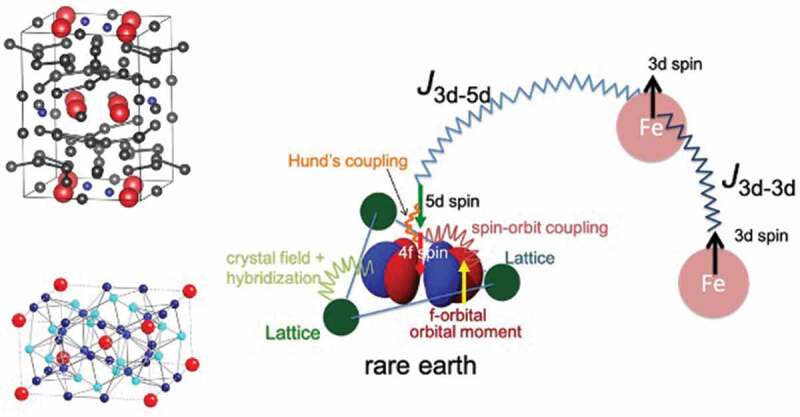

## 1. Introduction

Ever since Strnat and Hoffer have developed YCo${\;}_{5}$ [1], rare-earth transition-metal (R-T) compounds form a class of hard-magnetic compounds. Subsequently, they developed SmCo_5_ [2] which has extremely high magnetocrystalline anisotropy arising from the Sm-4f electrons. The Sm-Co-based magnets had been the strongest magnets in the 1970s before Sm_2_Co_17_, which has higher magnetization than SmCo_5_, was developed as a main phase. In 1982, the NdFeB-based magnet (neodymium magnet) was invented, in which Nd_2_Fe_14_B is the main phase [3]. This opened the era of iron-based rare-earth magnets. Several years later, iron-rich phases having the ThMn_12_ structure (RFe_12_-type compounds) [4–7] and Sm_2_Fe_17_N_3_ [8,9] came out.

**Figure 1.**
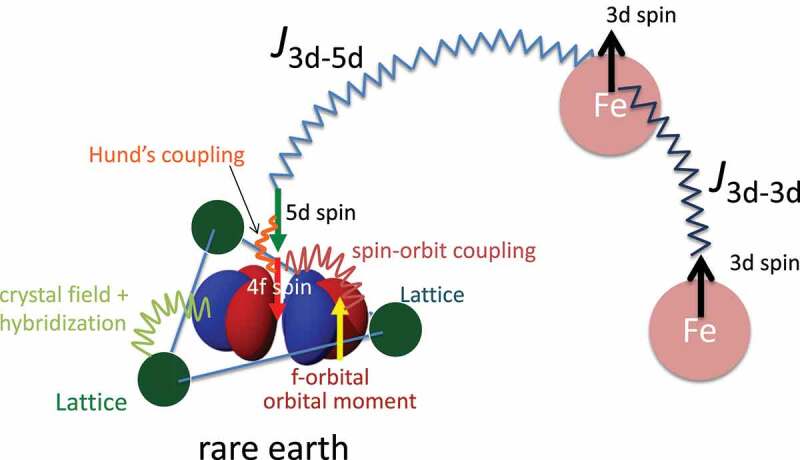
Magnetism in a rare-earth magnet compound

The main components of the rare-earth magnets are transition metals (Fe and/or Co) and rare-earth elements (e.g. Nd and Sm). The idea behind this combination is that high transition-metal content leads to high saturation magnetization and high Curie temperature, whereas rare earths are source of high magnetocrystalline anisotropy which is essential to achieve high coercivity (Figure 1). Therefore, search for stable phases of iron-rich rare-earth compounds has been an important issue in the development of high-performance permanent magnets. Research activity in this direction has been high in the past decade, as the performance of the NdFeB-based magnet approaches its theoretical limit by the improvement of microstructure over decades. In particular, the RFe_12_-type compounds (R= rare earth) attract much attention because of its high Fe content. It should be noted among many trials that Hirayama et al. have successfully synthesized a NdFe_12_N film [10], stimulated by first-principles calculation [11]. They measured its intrinsic magnetic properties, and found that it has higher saturation magnetization and higher anisotropy field at room temperature, and also higher Curie temperature than Nd_2_Fe_14_B.

In this article, we review theoretical works on rare-earth magnets. We start with discussing computational techniques based on first-principles calculation in Sec.2. In Sec.3, magnetism and stability of rare-earth magnet compounds are discussed. We then demonstrate how data-driven approaches help us optimize the chemical composition of a multinary compound in Sec.4. The paper is concluded in Sec.5.

## 2. First-principles calculation

Density functional theory (DFT) [12,13] is a standard first-principles calculation method. In DFT, the total energy of a many-electron system is expressed as a functional of electron density n(r):

[1] $$E[n]=T_{s}[n]+\frac{e^{2}}{2}\int \frac{n(r)n(r^{\prime })}{|r-r^{\prime }|}d^{3}rd^{3}r{\;}^{\prime }+E_{xc}[n]+\int v_{ext}(r)n(r)d^{3}r+\frac{1}{2}\sum_{I\neq J}\frac{Z_{I}Z_{J}e^{2}}{|R_{I}-R_{J}|}\;.$$

Here, the first term of the right side, $T_{s}\equiv -\frac{\hbar^{2}}{2m}\sum_{j}\langle \psi_{j}|\nabla^{2}|\psi_{j}\rangle$, is the kinetic energy of a *fictitious* non-interacting electron system, the second term is the electrostatic term between electrons (Hartree term), the third term is the exchange-correlation term, the fourth term is the electron–ion interaction, and the last term is the ion–ion interaction. The $E_{xc}$ contains all the electron–electron interaction effects other than the Hartree term. The exact expression of it is not known, and either the local density approximation (LDA) or the generalized gradient approximation (GGA) is frequently adopted. The ground-state electron density is obtained by minimizing eq.(1). This is achieved by solving the Kohn-Sham equation self-consistently:

[2] $$\left\{-\frac{\hbar^{2}}{2m}\nabla^{2}+v_{eff}(r)\right\}\psi_{j}(r)=\epsilon_{j}\psi_{j}(r)\;,$$

[3] $$v_{eff}(r)=e^{2}\int \frac{n(r^{\prime })}{|r-r^{\prime }|}d^{3}r{\;}^{\prime }+\frac{\delta E_{xc}[n]}{\delta n(r)}+v_{ext}(r)\;,$$

[4] $$n(r)=\sum_{j}|\psi_{j}(r){|}^{2}\;.$$

**Figure 2.**
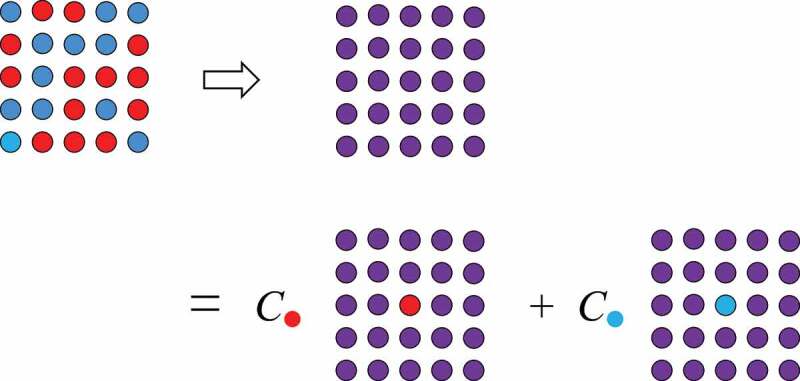
In the coherent potential approximation, random alloy is replaced with an impurity problem

The electronic density of states, electron and spin densities and magnetic moments are obtained from the eigenvalues $\left\{\epsilon_{j}\right\}$ and eigenfunctions $\left\{\psi_{j}\right\}$. Inserting the electron density n(r) in eq.(1), the total energy is obtained. The structure is optimized so that the total energy is minimized. To deal with magnetic systems, generalization of DFT for spin-polarized systems was developed [14,15]. The Curie temperature ($T_{C}$), crystal-field coefficients, spin-wave dispersion and exchange stiffness are obtained as a post-process calculation using the self-consistent solution [16–20]. A common scheme for evaluating the $T_{C}$ from first-principles is the following. One computes the intersite exchange couplings $J_{ij}$ by the Liechtenstein method [21], from which a classical Heisenberg model is derived. The Curie temperature is evaluated by solving the model using e.g. mean-field approximation or Monte Carlo simulation. In the mean-field approximation, the $T_{C}$ is overestimated. Non-stoichiometric systems are hard to treat in a conventional electronic-structure framework, because a large unit cell is required when periodicity is broken. In the Korringa-Kohn-Rostoker (KKR) method in the Green function theory [22,23], however, coherent potential approximation (CPA) [24] is available. In the CPA, a disordered system, e.g. random alloy, is mapped onto a single-impurity problem in an effective medium with an energy-dependent self-energy. The effective medium is determined self-consistently in such a way that the effective-medium Green’s function is equal to the configuration averaged Green’s function of the impurity system (Figure 2).

**Figure 3.**
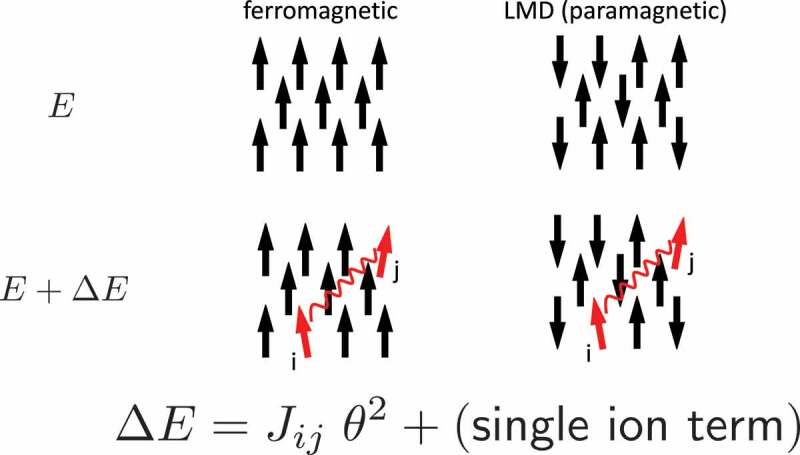
Intersite exchange coupling for the ferromagnetic state and LMD (local moment disorder) state

**Figure 4.**
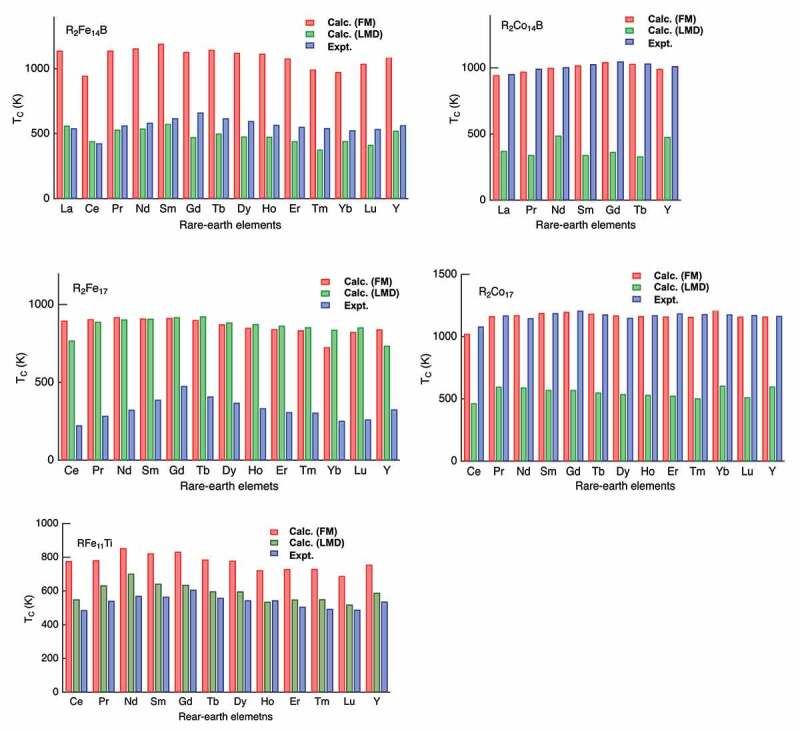
Curie temperatures ($T_{C}$) of $R_{2}$Fe${\;}_{14}$B, $R_{2}$Co${\;}_{14}$B, $R_{2}$Fe${\;}_{17}$, $R_{2}$Co${\;}_{17}$ and RFe${\;}_{11}$Ti

In general, careful treatment of electron interaction is required for quantitative description of magnetism, because magnetism is essentially outcome of quantum many-body effects. The validity and limitations of above schemes have been studied through applications to wide range of materials [16]. The overall trend of magnetism in 3d metals is well captured by the method. It should be noted that the experimentally observed Slater-Pauling curve for both the magnetization and Curie temperature [25] of 3d transition metal alloys is reproduced by first-principles calculation. As an extension, the magnetization and the Curie temperature of hypothetical atoms arranged in the bcc structure were calculated as functions of atomic number and the lattice constant [26]. It was concluded that plausible upper limits of the saturation magnetization and $T_{C}$ are 2.7 T and 2000 K, respectively. Conventional first-principles schemes also reasonably reproduce the magnetization of rare-earth magnet compounds. Calculated magnetizations in $R_{2}$Fe${\;}_{14}$B and $R_{2}$Co${\;}_{14}$B for a series of R are in good agreement with the experiment [27]. The Curie temperatures of $R_{2}$Co${\;}_{14}$B are also quantitatively reproduced, while those of $R_{2}$Fe${\;}_{14}$B are systematically overestimated, although dependence on R is well captured [27]. Here, the $T_{C}$s were evaluated in the mean-field approximation. Therefore, we need to be careful when we analyze the results. Apart from this problem, the situation for $T_{C}$ is indeed complicated. Local moments in a magnetic metal fluctuate relatively slowly on the time scale of the other electronic degrees of freedom. The exchange coupling between the local moments $J_{ij}$s are normally evaluated for the ground state (Figure 3). This assumption is not necessarily valid near $T_{C}$. When we use $J_{ij}$s for the local-moment-disorder (LMD) state [28], which is also called as disordered local moment (DLM) [29], $T_{C}$ is significantly changed. Figure 4 shows $T_{C}$ of typical rare-earth magnet compounds. In $R_{2}$Fe${\;}_{14}$B, the $T_{C}$s are systematically overestimated if we use $J_{ij}$s for the ground state, whereas the agreement is much better when $J_{ij}$s for LMD are used. In $R_{2}$Co${\;}_{14}$B, the $T_{C}$s for the ground-state $J_{ij}$s are in good agreement with the experiment, whereas LMD $J_{ij}$s lead to substantial underestimation of $T_{C}$s. In $R_{2}$Fe${\;}_{17}$, both $J_{ij}$s result in too high $T_{C}$s, while $T_{C}$s in $R_{2}$Co${\;}_{17}$ are well reproduced by using $J_{ij}$s for the ground state. The $T_{C}$s in RFe${\;}_{11}$Ti agree well when $J_{ij}$s for LMD are adopted. These complicated dependence on the crystal structure and the transition-metal element may originate from difference in spin fluctuation near the magnetic transition, and further analysis is needed to solve the problem.

A direct method to evaluate the magnetocrystalline anisotropy energy (MCA) is the total-energy-difference method. In this method, the total energy when the magnetization is along the easy axis is compared to that along the hard axis. The MCA can be also evaluated from the band energy using the force theorem [30–32] based on the perturbation theory when the spin–orbit interaction is weak or moderate. On the other hand, 4f electrons in rare-earth (RE) elements are hard to treat. There is no established approximation that is quantitatively accurate enough, and at the same time computationally cheap for practical use. Development of first-principles methods is an important issue. Trials using e.g. self interaction correction (SIC) [33,34] and dynamical mean-field theory (DMFT) [35,36] are underway. A conventional method for the MCA energy in rare-earth magnet compounds is based on the crystal field theory [37,38]. In this method, the crystal-field (CF) coefficients are computed by expanding the Kohn-Sham effective potential $v_{eff}$ in eq.(3) by real spherical harmonics. The magnetocrystalline anisotropy constant is evaluated from the CF parameter $A_{2}^{0}$ as

[5] $$K_{1}=-3J(J-1)\alpha_{J}\langle r^{2}\rangle A_{2}^{0}n_{R}\;,$$

where J is the total angular momentum, $\alpha_{J}$ is the first Stevens factor, $\left\langle r^{2}\right\rangle$ is the spread of RE-4f orbitals, and $n_{R}$ is the rare-earth concentration.

Theoretical methods to go beyond these conventional schemes have been continuously developed. Especially, finite-temperature magnetism is a recent hot topic. The local moments are not perfectly aligned at finite temperature, but they are distributed in different directions by thermal fluctuation. First-principles calculation of temperature-dependent magnetic properties based on LMD (DLM) picture has a long history [16]. In the field of hard magnets, the DFT-DLM method has been applied to finite-temperature magnetization of YCo${\;}_{5}$ [39], and then RCo${\;}_{5}$ (R=Y-Lu), where the R-4f electrons were treated in SIC with orbital polarization correction [40]. The method was also applied to calculating the magnetic anisotropy [41,42].

Another approach is based on an effective Hamiltonian. A simple procedure is a classical spin-model approach [43]. The method, combined with first-principles calculation, was applied to Nd${\;}_{2}$Fe${\;}_{14}$B [17,18,44,45]. Recent activities in this approach are reviewed by Miyashita et al. in Refs. [46,47]. Quantum effects of the R-4f electrons can be investigated by solving a CF Hamiltonian. Sasaki et al. studied the magnetic anisotropy constants in Nd${\;}_{2}$Fe${\;}_{14}$B using an empirical CF Hamiltonian [48]. They could reproduce temperature dependence of $K_{1}$ and $K_{2}$, and discussed interplay between the exchange field and CF strength at finite temperature. Direct expression of the magnetic anisotropy constants based on CF theory was derived by Miura et al. [49,50]. Yoshioka et al. have evaluated the CF parameters in $R_{2}$Fe${\;}_{14}$B [51], SmFe${\;}_{12}$ [52], and Sm${\;}_{2}$Fe${\;}_{17}$N${\;}_{x}$ [53] by first-principles calculation. They studied finite-temperature magnetism using CF Hamiltonian, with emphasis on J-mixing effects.

As a more accurate treatment of the R-4f electrons, a DFT+DMFT method for rare-earth magnet compounds has been developed [54]. In this method, the system is mapped onto an interacting impurity problem, and the R-4f states at the impurity site are solved self consistently in the Hubbard-I approximation. The CF parameters are deduced from the obtained energy levels. The method has been applied to RFe${\;}_{12}$(N) [54] and RCo${\;}_{5}$ [55], and unexpectedly large higher-order CF parameters were obtained. Note that the effective interaction parameters (U and J) are treated as adjustable parameters in these calculations. First-principles methods for evaluating these parameters are actively studied [56–60].

Finally, we note that aforementioned calculations were carried out for fixed structures. However, it was pointed out by Tanaka and Gohda that inclusion of phonon affects the Curie temperature of bcc Fe [61,62]. Impact of phonon on the magnetism in rare-earth magnet compounds is an open question.

## 3. Magnetism and stability of Nd_2_Fe_14_B and R Fe_12_

When iron-based magnets beyond the Sm_2_Co_17_-based magnet were anticipated in the 1970s, Sm_2_Fe_17_ was considered to be a potential main phase of a new magnet. However, $T_{C}$ of Sm_2_Fe_17_, or more generally that of $R_{2}$Fe_17_, is too low. One possible cause for the low $T_{C}$ is short bond length between Fe(6*c*)-Fe(6*c*) (dumbbell irons). (Crystal structure data are presented in Appendix A). This, in turn, means that the ferromagnetism could be strengthened by stretching the bond. Based on this idea, Sagawa added boron to a Nd-Fe system, expecting that a small element would expand the volume of Nd_2_Fe_17_ and lead to strong ferromagnetism [63]. This is how he succeeded in developing the NdFeB-based magnet. Later on, Kanamori discussed the role of boron in Nd_2_Fe_14_B from the viewpoint of electronic states [64,65]. He pointed out that boron suppresses the local magnetic moments of neighboring Fe atoms by the chemical effect. This is called cobaltization. The cobaltized Fe atoms make the local magnetic moments of surrounding Fe atoms larger, which would result in the increase of the total magnetic moment of the compound.

In order to study the effect of the bond length between dumbbell irons on the Curie temperature, Fukazawa et al. evaluated intersite exchange couplings ($J_{ij}$) in Sm_2_Fe_17_ by first-principles calculation [66]. They found that dumbbell irons are ferromagnetically coupled with large $J_{ij}$ of 35 meV. Moreover, the $J_{ij}$ decreases with increasing the bond length. By elongating the bond from 2.4 Å to 2.6 Å, the calculated Curie temperature does increase, but only slightly by 2%. On the other hand, Dam et al. studied the Curie temperature by a completely different approach [67–69]. They constructed a machine-learning model by the kernel-ridge regression using experimental $T_{C}$ data for 101 R-T compounds. They used 27 descriptors which can be classified into three categories, that is, atomic properties of T (atomic number, covalent radius, ionization potential, etc.), atomic properties of R, and structural information (concentration of T, nearest distance between T-T etc.). Subgroup relevance analysis clarified that the most important and the only strongly relevant descriptor among the 27 descriptors is the rare-earth concentration, whereas the T-T distance is not significantly important. These results contradict the naive expectation mentioned above.

Tatetsu et al. studied the role of B in Nd_2_Fe_14_B by first-principles calculation [70]. They calculated the magnetizations of Nd_2_Fe_14_B, Nd_2_Fe_14_ and Nd_2_Fe_14_B_0_. Here, Nd_2_Fe_14_B_0_ denotes a hypothetical compound in which B is removed from Nd_2_Fe_14_B with fixing its structure, whereas the structure is optimized in Nd_2_Fe_14_. It is found that the local magnetic moments at the Fe(16$k_{1}$) and Fe(4e) sites are smaller in Nd_2_Fe_14_B than in Nd_2_Fe_14_B_0_. These sites are the nearest and second neighbors of B. This result is in accordance with the concept of cobaltization. The local magnetic moments of other Fe sites, which can be regarded as the neighbors of the cobaltized Fe sites, are slightly enhanced. This confirms the importance of the chemical effect on the magnetic moments, as proposed by Kanamori. (Effects of typical elements on the magnetic moment was precisely discussed for a related compound, NdFe${\;}_{11}$TiX with X = B, C, N, O, F in Ref [71].) The total magnetic moment is smaller in Nd_2_Fe_14_B than in Nd_2_Fe_14_B_0_. This means that the chemical effect does not enhance the magnetic moment of the whole system. By comparing the magnetic moment in Nd_2_Fe_14_B_0_ with that in Nd_2_Fe_14_, it is found that the magnetovolume effect enhances the total magnetic moment, whereas the magnetization (per volume) is smaller. It is also found that B has an essential role in stabilizing the Nd_2_Fe_14_B structure by comparing the total energy of Nd_2_Fe_14_B with that of Nd_2_Fe_17_B.

The *R*Fe_12_-type compounds in the ThMn_12_ structure have been revisited recently from both theoretical and experimental sides [72,73]. One problem is that these compounds are thermodynamically unstable, so that their bulk samples cannot be synthesized. The structure is stabilized by partially substituting another element for Fe sites. However, introduction of the stabilizing elements normally deteriorates the magnetization, hence search for an element that stabilizes the structure while keeping high magnetization is an important issue.

Harashima et al. have carried out first-principles calculation of NdFe_11_M for M=Ti, V, Cr, Mn, Fe, Co, Ni, Cu, Zn [74]. It is found that the ThMn_12_ structure is stabilized when M=Ti occupies the 8i site, while Ti suppresses the magnetic moment significantly. This in turn means that the magnetic moment is enhanced if Ti content can be reduced [11,75]. Both the formation energy and the magnetic moment are sensitive to the choice of the M element. For early transition metal (M=Ti-Mn), the magnetic moment is substantially smaller than NdFe${\;}_{12}$. The change of the moment in this region is qualitatively explained by Friedel’s concept of virtual bound state. The magnetic moment is larger for M=Co, Ni, Cu and Zn. In particular, the magnetic moment of NdFe_11_Co is comparable to that of NdFe_12_. Cobalt also enhances the stability of the ThMn_12_ structure and $T_{C}$. (In the low concentration region, Cr more efficiently enhances $T_{C}$ than Co [76].)

The formation energy of the RFe${\;}_{12}$-type compounds also depends on R [77]. It has a strong correlation with the atomic radius of the R element, $r_{R}$. First-principles calculation clarified that the formation energy with respect to simple substances becomes lower as $r_{R}$ decreases. When the Th_2_Zn_17_ structure is taken as a reference system, the formation energy shows a minimum at $r_{R}≃1.75$Å, which corresponds to R=Dy. This implies that SmFe_12_ is more stable than NdFe_12_, and partial substitution of other R elements such as Y, Gd and Zr for Sm will have positive effect on the stability of the ThMn_12_ structure. The formation energy also depends on the valency of the R ion, and tetravalent Ce is a promising stabilizer of the ThMn_12_ structure [78].

The interstitial dopant at the 2b site also affects the magnetic properties [71]. When the atomic number Z of the dopant X is hypothetically changed, the magnetic moment increases by changing from Z=6 to Z=7. This is explained by the change in the density of states. The energy level of the X-2p state, hybridized with the Fe-3d state, is pulled down with increasing Z. The band is partially filled in the majority spin channel for Z=7 more than Z=6, which results in the increase of the magnetic moment. The hybridized state contains not only X-2p and Fe-3d character but also Nd-5d character. Since the Nd-5d spin is anti-parallel to the total moment, its magnetic moment is reduced as the level of the hybridized state is downshifted. The change in the Nd-5d moment is correlated with the exchange coupling between Nd and surrounding Fe sites [79], which is a key quantity for the temperature dependence of the anisotropy field [80].

We note here a difference between B and N in R-T compounds. Interstitial nitrogen enhances both the Curie temperature and magnetization significantly [9]. It also has a big impact on the magnetocrystalline anisotropy [81] through the change in electron density around the rare-earth atoms [11,82]. The induced charge is, however, so weak to form a chemical bonding that structural phase transition does not occur. In this sense, the term *nitrogenation* instead of nitride is often used for nitrogen doping. In contrast, boron induces structural transformation in R-T systems as mentioned above.

## 4. Materials informatics

Materials informatics has been rapidly growing in the past ten years. From an early stage, first-principles calculation has played a main role. High throughput calculation for various structures and chemical compositions were carried out, and databases, e.g. Materials Project [83], Open Quantum Materials Database (OQMD) [84], AFLOW [85,86], were developed. Machine learning was introduced for efficiently predicting and understanding materials properties from available materials data.

Research activity in hard magnetic materials is gradually increasing recently [87]. Körner et al. have carried out high-throughput calculations of ThMn_12_-type [88], YNi_9_In_2_-type [89], and 1–13-X compounds [90]. Nieves et al. have been developing a database for rare-earth free/lean permanent magnets [91]. Independently, Sakurai et al. developed a database for rare-earth-free magnetic materials [92].

**Figure 5.**
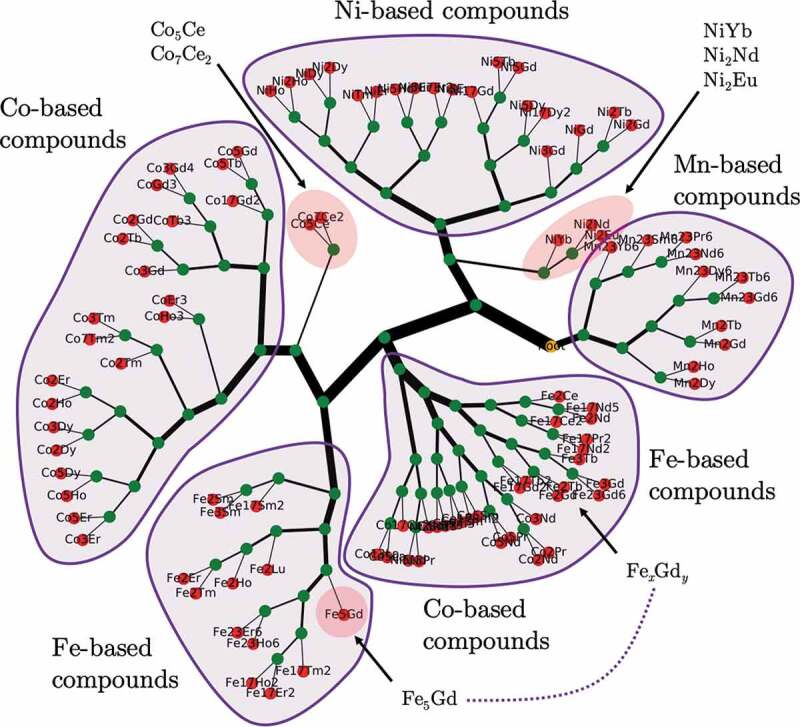
Hierarchical clustering of rare-earth transition-metal compounds by obtained dissimilarity voting machine using experimental Curie temperature data. From Ref [68]

There are several papers published on machine learning of magnetic materials. One is kernel-ridge regression of the experimental $T_{C}$s in about 100 rare-earth transition-metal compounds, as mentioned in the previous section [67]. The distance between materials can be measured by the kernel regression. Using this property, Nguyen et al. proposed dissimilarity voting machine based on ensemble learning, and applied the method to the rare-earth transition-metal compounds (Figure 5) [93]. Nelson and Sanvito compared the performance of several different regression models using experimental $T_{C}$ data for about 2500 known ferromagnetic compounds, and showed that the best model predicts $T_{C}$ with an accuracy of ∼50 K [94]. Long et al. applied random forrest for classifying ferromagnetic and antiferromagnetic compounds and predicting the Curie temperature [95].

**Figure 6.**
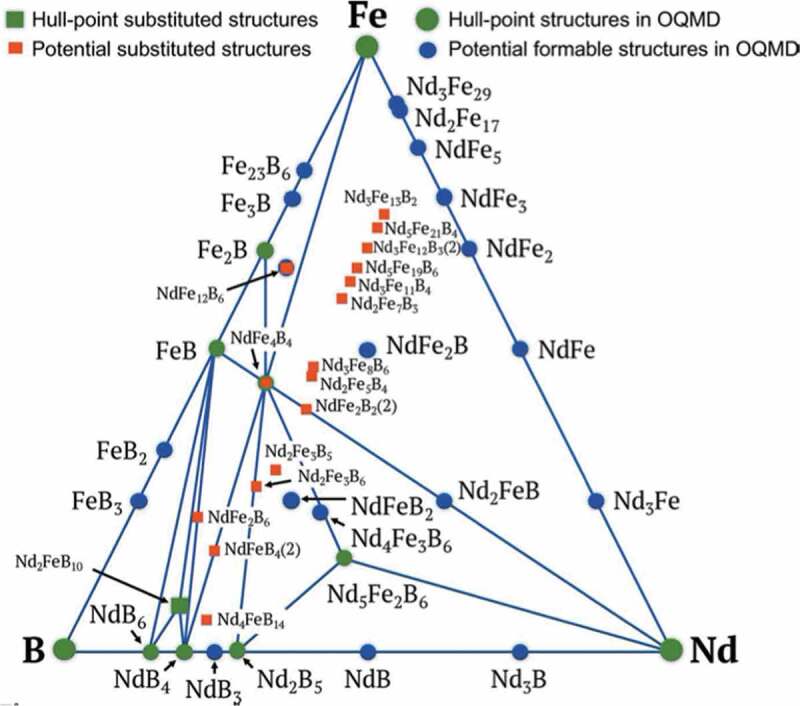
Potentially formable phases of Nd-Fe-B systems obtained by theoretical exploration. See Ref [98]. for the comparison between direct screening by first-principles calculation and virtual screening using machine learning. From Ref [98]

Design of a good descriptor is an important issue in materials informatics. Pham et al. proposed a novel representation of a material, called Orbital Field Matrix (OFM), based on Voronoi diagram [96,97]. In OFM, a local structure in a material is represented by a matrix using the electron configuration of the central atom in a Voronoi polyhedron and that of neighboring atoms. The performance of OFM was examined for the formation energy and magnetic moment of about 4000 transition-metal compounds. They carried out machine-learning-aided screening of Nd-Fe-B systems using OFM as a descriptor, and found several potentially formable phases (Figure 6) [98]. Halder et al. predicted magnetic properties of Ce-based 2–17-X systems using a combination of first-principles calculation and machine learning [99].

**Figure 7.**
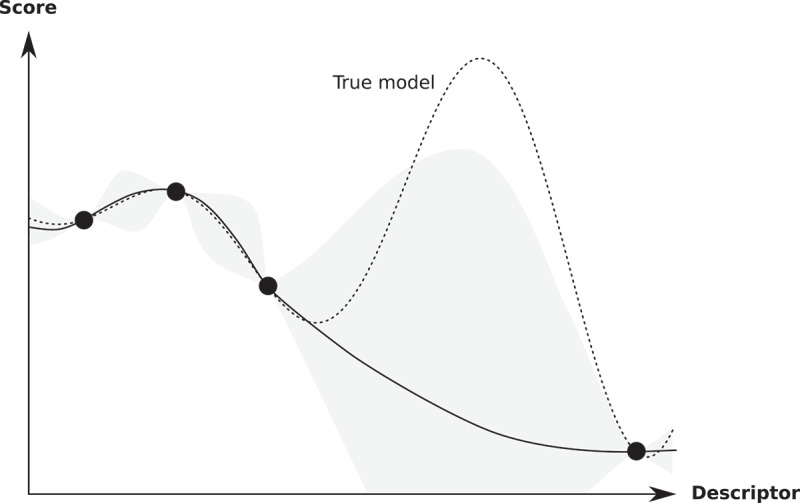
Schematic of Bayesian optimization. Already sampled points are shown by closed circles. In the Bayesian optimization, the next candidate is selected by taking account of the uncertainty of a model (shaded area) in addition to the mean value (solid line) of a prediction model obtained by the sampled data. From Ref [100]

In what follows, we describe two data-driven approaches in more detail. One is the Bayesian optimization, and the other is data assimilation. Chemical substitution is a common procedure to improve magnetic properties. As the number of dopants increases, however, search space becomes wider exponentially, which makes optimization of chemical composition difficult. Bayesian optimization is a powerful tool in such a situation. Suppose we have four data, as shown in Figure 7. Here, the horizontal axis is numerical representation of a material, called descriptor. In our case, it denotes chemical composition. A question is which point we will try next to get a high score. If the descriptor is one dimension, we may just change the value of the descriptor slightly and repeat the process many times. However, this scheme does not work when the descriptor is high dimension. In the Bayesian optimization, the next candidate is selected by taking account of the uncertainty of a model in addition to the mean value. Figure 7 is a typical situation. In this case, the highest score is not selected if we consider the mean value only, and exploration based on uncertainty is important. In Ref [100], the Bayesian optimization has been adopted to optimize the chemical composition of the RFe${\;}_{12}$-type compound. More precisely, the composition of $(R_{1-\alpha },Z_{\alpha })($Fe${\;}_{1-\beta }$Co${\;}_{\beta }{)}_{12-\gamma }$Ti${\;}_{\gamma }$ (R=Nd, Sm, Y; Z=Zr, Dy) was optimized in terms of the formation energy, saturation magnetization and Curie temperature. The success rate of finding top 10 systems out of 3630 compositions within 50 trials was determined by 1000 independent sessions. As a result, it is found that the Bayesian optimization has high success rate (>95%) for all the three target variables if the descriptor is appropriately chosen, which is by far higher than that of random sampling, 12.9%.

**Figure 8.**
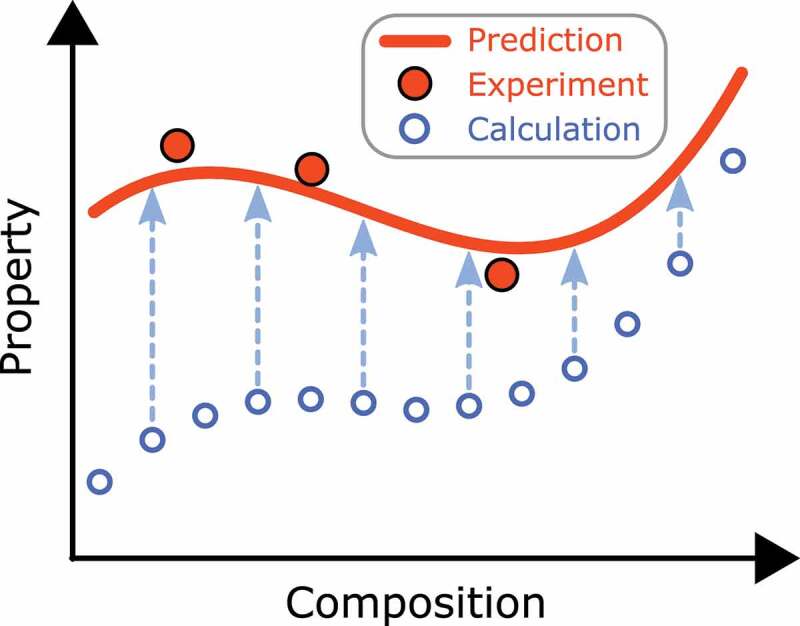
Schematic illustration of data-assimilation method

**Figure 9.**
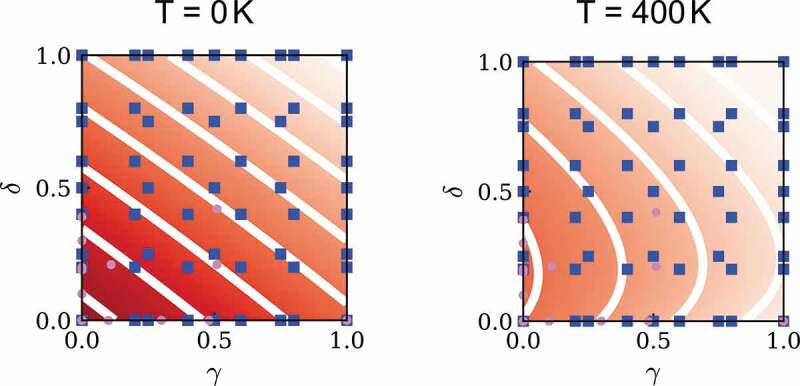
Magnetization of (Nd${\;}_{1-\gamma }$Ce${\;}_{\gamma }$)${\;}_{2}$(Fe${\;}_{1-\delta }$Co${\;}_{\delta }$)${\;}_{14}$B at 0 K and at 400 K. At 0 K, the magnetization is the highest at (δ,γ) = (0,0), and monotonically decreases with increasing δ and γ. At 400 K, the magnetization increases with increasing Co concentration for small δ, and turns to decrease for further increasing δ. From Ref [101]

A more brute-force method for optimizing composition is high-throughput first-principles calculation using supercomputers. A bottleneck of this approach is that first-principles calculation includes a theoretical error originated from approximations contained in the calculation, thereby computational data systematically deviates from true values, although material dependence is captured. For example, as mentioned above, the Curie temperature is overestimated in the mean-field approximation. On the other hand, the number of available experimental data is often too small, hence an accurate prediction model is hard to construct by solely using the experimental data. If we utilize both the computational and experimental data, however, an accurate model can be derived. Harashima et al. have formulated a data-assimilation method in which a small number of experimental data are integrated with a large number of first-principles calculation data (Figure 8) [101]. The method can be applicable for data sets including missing values. The method was applied to estimate the finite-temperature magnetization of partially substituted (Nd, Pr, La, Ce)${\;}_{2}$(Fe, Co, Ni)${\;}_{14}$B. In this application, experimental data were collected for 119 samples. The magnetization was measured for 3–7 temperatures in each sample, from which magnetization at 0 K, $\mu_{0}M_{0}$, and $T_{C}$ were evaluated using Kuz’min’s formula [102]. Independently, first-principles calculations of $\mu_{0}M_{0}$ and $T_{C}$ for 2869 compositions were carried out. By adopting the data-assimilation method, accurate models for $\mu_{0}M_{0}$ and $T_{C}$ were obtained. Using Kuz’min’s formula in the final step, the magnetization at arbitrary composition of the eight-component system at arbitrary temperature was evaluated. Figure 9 shows the magnetization at 0 K and 400 K. We can see that the magnetization varies monotonically with increasing Ce and Co concentrations at 0 K, whereas the magnetization is enhanced by partial substitution of Co at 400 K.

## 5. Concluding remarks

Although first-principles calculation is a powerful tool for the development of magnetic materials, there are remaining problems to be fixed. As we discussed, accuracy of the current theoretical methods is still limited. Magnetism is essentially outcome of quantum many-body effects. In particular, treatment of rare-earth 4f electrons is to be improved. Quantitative description of magnetism and stability at finite temperature is also under development.

Another direction is materials exploration. First-principles calculation, coupled with machine-learning techniques, has developed rapidly in the last decade. Machine learning is particularly efficient in the optimization of chemical composition. When completely new material is concerned, however, current techniques are not sufficient. Methods for crystal structure prediction and finding new phases is actively investigated, and application to magnetic materials is a hot topic [98,103]. Data-driven approaches for thermodynamic properties, such as phase diagram for multinary compounds, are also to be developed.

## Appendix A. Optimized structure in DFT-GGA

The space group of the Nd_2_Fe_14_B structure is P4_2_/mnm, No.136. The calculated lattice constants are *a* = 8.791Å,*C* = 12.141Å [70]. The inner coordinates are presented in Table 1.

**Table 1.** The inner coordinates for Nd_2_Fe_14_B [70]

atom	site	*x*	*y*	*z*
Nd	4*f*	0.266	0.266	0
Nd	4*g*	0.142	−0.142	0
Fe	16*k_1_*	0.225	0.567	0.127
Fe	16*k_2_*	0.037	0.360	0.177
Fe	8*j_1_*	0.098	0.098	0.205
Fe	8*j_2_*	0.317	0.317	0.246
Fe	4*e*	1/2	1/2	0.114
Fe	4*c*	0	1/2	0
B	4*g*	0.375	−0.375	0

The space group of Sm_2_Fe_17_ in the Th_2_Zn_17_ structure is R$\overset{ˉ}{3}$m, No.166. The calculated lattice constants are *a* = 8.526Å,*c* = 12.455Å [77]. The inner coordinates are presented in Table 2.

**Table 2.** The inner coordinates for Sm_2_Fe_17_ [77]

atom	site	*x*	*y*	*z*
Sm	6*c*	0	0	0.3407
Fe	18*f*	0.2927	0	0
Fe	18*h*	0.5006	−0.5006	0.1571
Fe	9*d*	1/2	0	1/2
Fe	6*c*	0	0	0.0965

The space group of Sm_2_Fe_17_N_3_ is R$\overset{ˉ}{3}$m, No.166. The calculated lattice constants are *a* = 8.675Å,*c* = 12.556Å. The inner coordinates are presented in Table 3.

**Table 3.** The inner coordinates for Sm_2_Fe_17_N_3._

atom	site	*x*	*y*	*z*
Sm	6*c*	0	0	0.3434
Fe	18*f*	0.2822	0	0
Fe	18*h*	0.5052	−0.5052	0.1528
Fe	9*d*	1/2	0	1/2
Fe	6*c*	0	0	0.0945
N	9*e*	1/2	0	0

The space group of SmFe_12_ in the ThMn_12_ structure is I4/mmm, No.139. The calculated lattice constants are*a* = 8.497Å,*c* = 4.687Å [75]. The inner coordinates are presented in Table 4.

**Table 4.** The inner coordinates for SmFe_12_ [75]

atom	site	*x*	*y*	*z*
Sm	2*a*	0	0	0
Fe	8*f*	1/4	1/4	1/4
Fe	8*i*	0.3588	0	0
Fe	8*j*	0.2696	1/2	0
